# Nuclear medicine in the management of patients with heart failure: guidance from an expert panel of the International Atomic Energy Agency (IAEA)

**DOI:** 10.1097/MNM.0000000000000143

**Published:** 2014-07-03

**Authors:** Amalia Peix, Claudio Tinoco Mesquita, Diana Paez, Carlos Cunha Pereira, Renata Felix, Claudia Gutierrez, Rodrigo Jaimovich, Barbara Maria Ianni, Jose Soares, Pastor Olaya, Ma. Victoria Rodriguez, Albert Flotats, Raffaele Giubbini, Mark Travin, Ernest V. Garcia

**Affiliations:** a Department of Nuclear Sciences and Applications, Division of Human Health, Section of Nuclear Medicine and Diagnostic Imaging, International Atomic Energy Agency, Vienna, Austria; b Instituto de Cardiología y Cirugía Cardiovascular, Havana, Cuba; c Hospital Universitário Antonio Pedro, Universidade Federal Fluminense (UFF), Niteroi; d Quanta Diagnostico Nuclear, Curitiba; e Instituto Nacional de Cardiologia, Rio de Janeiro; f Instituto do Coração, Universidade de São Paulo, São Paulo, Brazil; g Fundación Cardioinfantil, Instituto de Cardiología, Bogotá; h Fundación Clínica Valle Del Lili, Cali, Colombia; i Hospital Clínico, Facultad de Medicina, Pontificia Universidad Católica de Chile, Santiago, Chile; j Hospital General de Coro, Coro, Venezuela; k Universitat Autònoma de Barcelona, Department of Nuclear Medicine Hospital de la Santa Creu i Sant Pau, Barcelona, Spain; l Cattedra e U.O. di Medicina Nucleare, Università e Spedali Civili, Brescia, Italy; m Montefiore Medical Center, Yeshiva University, New York, New York; n Department of Radiology, Emory University Hospital, Emory University School of Medicine, Atlanta, Georgia, USA

**Keywords:** coronary artery disease, dilated cardiomyopathy, gated myocardial perfusion SPECT, left ventricular dyssynchrony, MIBG, PET imaging, heart failure

## Abstract

Heart failure is increasing worldwide at epidemic proportions, resulting in considerable disability, mortality, and increase in healthcare costs. Gated myocardial perfusion single photon emission computed tomography or PET imaging is the most prominent imaging modality capable of providing information on global and regional ventricular function, the presence of intraventricular synchronism, myocardial perfusion, and viability on the same test. In addition, ^123^I-mIBG scintigraphy is the only imaging technique approved by various regulatory agencies able to provide information regarding the adrenergic function of the heart. Therefore, both myocardial perfusion and adrenergic imaging are useful tools in the workup and management of heart failure patients. This guide is intended to reinforce the information on the use of nuclear cardiology techniques for the assessment of heart failure and associated myocardial disease.

## Introduction

Heart failure (HF) is growing globally at epidemic proportions, causing considerable increases in disability and mortality as well as in healthcare costs 1,2. Coronary artery disease (CAD), diabetes mellitus, and hypertension are major etiological risk factors. HF affects more than 15 million people worldwide 2. Dilated cardiomyopathy (DCM) refers to a heterogeneous spectrum of myocardial diseases that are characterized by ventricular dilation and reduced myocardial contractility. Once patients become symptomatic, the prognosis is relatively poor, with 25% mortality at 1 year and 50% mortality at 5 years 3. Among the causes of acquired DCM in Latin America, Chagas cardiomyopathy is one of the most common, with a prevalence of ∼24 million 4 of HF in areas where the disease is endemic. Given the morbidity and mortality from HF, as well as the considerable resources that are used to diagnose and treat these patients, appropriate diagnosis and prognosis assessment are vital.

Gated myocardial perfusion ^99m^Tc single photon emission computed tomography (SPECT) or PET imaging (MPI) is the most prominent imaging modality capable of providing, in a reproducible manner, information on global and regional ventricular function, the presence of intraventricular synchronism, myocardial perfusion, and viability on the same test. In addition, ^123^I-mIBG scintigraphy is the only imaging technique approved by various regulatory agencies able to provide information regarding the adrenergic function of the heart. Therefore, both adrenergic imaging and MPI are useful tools in the workup and management of HF patients.

Results from a worldwide meta-analysis of all pertinent clinical trials up to 2007 suggest that ∼1–3% of all patients discharged alive after hospitalization for HF and 15–20% of all patients seen in HF clinics meet cardiac resynchronization therapy (CRT) eligibility criteria 5; thus, all of these patients could be candidates for MPI dyssynchrony analysis. As about half of these numbers also meet the criteria for implantable cardiac defibrillator (ICD) implantation 5, it is also prudent to consider imaging these patients with ^123^I-mIBG as an adjunct to their risk stratification for treatment selection. The specific number of HF patients who would benefit from undergoing these imaging techniques for a specific country is thus dependent on accurate statistics in terms of the total number of HF patients seen in clinics, which is a difficult number to obtain, depending on the country. It is estimated that HF affects 23 million people worldwide 6.

This guidance is a consensus statement from an international panel of nuclear medicine experts assembled by the International Atomic Energy Agency (IAEA). The initial purpose was to address how to use our techniques to help manage patients with Chagas disease, but the focus quickly transitioned to the topic of addressing the potential use of nuclear medicine techniques in all patients with HF. This guidance is intended to reinforce the information on the use of available nuclear cardiology techniques for the assessment of HF and associated myocardial disease. This article is not intended as an exhaustive review of all data on the subject of HF imaging but rather as a consensus statement of experts with knowledge on the existing evidence, mainly addressed to developing countries with limited resources. Because PET radiopharmaceuticals used in HF assessment are not widely available the panel focused on scintigraphic planar and SPECT procedures.

## Why should a heart failure patient be sent for nuclear imaging?

The prevalence of heart failure is increasing in both developed and developing countries. Ischemic cardiomyopathy is the most common etiology of chronic HF 1. The initial clinical management decision in HF patients is based on the differentiation of ischemic from nonischemic etiologies. Among a variety of diagnostic approaches including cardiac catheterization and several noninvasive imaging techniques, nuclear cardiology techniques such as MPI and ^123^I-mIBG imaging have an important role in the diagnostic workup and risk assessment of patients with HF.

### Myocardial perfusion imaging

Recent recommendations 7 and appropriate use criteria state that nuclear cardiology techniques are adequate in patients with new-onset or newly diagnosed HF 8. The main indication for MPI in the HF population is for the identification of patients who may benefit from therapy for myocardial ischemia and ventricular dysfunction, which includes medical treatment, revascularization, and resynchronization devices.

#### Cardiac resynchronization therapy

CRT is a biventricular pacemaker with a third electrode attached to the left ventricle to assist in resynchronizing the mechanical contraction of the left ventricular (LV). CRT has been shown to benefit some patients with end-stage HF, depressed left ventricular ejection fraction (LVEF) (<35%), and a wide QRS complex on the surface ECG (>120 ms 9 or even >150 ms 10), and is now standard treatment for HF 10. As electrical activation is often decoupled from the onset of mechanical contraction, the main indication for MPI in patients being considered for CRT is for the identification of LV mechanical dyssynchrony and for guiding the placement of the LV lead to the last viable contracting segment for optimal response (improvement of LV function) 11.

The specific reasons why a patient with heart failure should be referred to nuclear cardiology myocardial perfusion imaging:

1. To assess myocardial ischemia.
2. To assess LV global and regional viability.
3. To assess whether LV function is preserved, including regional wall motion and thickening, LVEF, LV volumes, and myocardial LV mass.
4. To assess LV eccentricity 12.
5. To assess LV intraventricular dyssynchrony 13,14.
6. To assess the last viable segment to contract in patients being considered for CRT 15.

In addition, assessing patients’ risk of cardiac death contributes to the determination of the probability of sudden death and the need for an ICD 16.

### ^123^I-mIBG imaging

Much evidence has accumulated to show that cardiac autonomic imaging, such as with ^123^I-mIBG, is a powerful risk stratification tool for patients with HF 17. In the setting of this condition, normal autonomic balance is disrupted, which is not only evidence of the severity of the condition but also an indicator of a worsening prognosis 17,18. With ^123^I-mIBG imaging, which is based on a false transmitter analog of norepinephrine, we can assess cardiac sympathetic innervation.

On the basis of worldwide experience, the EANM Cardiovascular Committee and the European Council of Nuclear Cardiology have recently proposed standardized methods 19, but more work needs to be done in this regard. The ideal imaging procedures, including patient preparation, medication holding, dosage of tracer, acquisition protocols, and quantitative methods, are evolving 20. At this time, planar ^123^I-mIBG imaging procedures are well established, although more work is needed for SPECT imaging and comparative analysis with MPI.

^123^I-mIBG availability is severely limited throughout the world, being actively used only in specific regions. Although available in Japan, Europe, and Brazil for a long time, ^123^I-mIBG has just been recently approved in the USA by the FDA for HF evaluation.

Multiple studies have shown that ^123^I-mIBG imaging, especially determination of the heart-to-mediastinum (H/M) ratio, very effectively separates high-risk from low-risk patients, regardless of LVEF and NYHA clinical conditions 17,21. In fact, patients with a normal H/M ratio have an excellent prognosis despite other abnormal cardiac parameters, such as LVEF and brain natriuretic peptide, and on multivariate analysis H/M has consistently been shown to provide independent incremental risk stratification power 17,21. However, risk stratification is useful only to the extent that it allows guidance of therapies that would improve patient outcome and well-being.

The specific reasons why a patient with heart failure should be referred to nuclear cardiology ^123^I-mIBG:

1. To assess ^123^I-mIBG cardiac uptake as given by the H/M ratio 18.
2. To use the H/M ratio to help risk stratify the patient 21.
3. To use the H/M ratio in risk stratification to help decide on a change of management decision. For example, the condition of an HF patient without ICD has changed and now ICD is being considered 22.

### Radiation burden

Concerns about medical exposure to ionizing radiation in cardiac patients have become heightened in recent years as a result of rapid growth in procedure volumes and the high radiation doses incurred from some procedures. Although several landmark epidemiological studies involving similar levels of radiation exposure show increased cancer risk, no strong data currently relate ionizing radiation specifically from cardiac imaging to increased risks of cancer 23. Important benefits of cardiac imaging for adequate patient management, such as correct diagnosis, accurate prognostication, and improvement of outcomes, should also be taken into account.

In general, radiation dose is less of a concern in elderly patients with HF because the risk of dying from heart disease is far greater than any radiation concern. Patients admitted to hospital with a diagnosis of cancer often have a longer survival compared with those with a diagnosis of HF. Prognosis in HF that requires hospitalization can be considered far worse than that of many common types of cancer. For example, in the Framingham cohort, 62 and 75% of men and 38 and 42% of women, respectively, died within 5 years of being diagnosed with HF 24. In comparison, 5-year survival rate from all cancers among men and women in the USA during the same period was ∼50% 24.

Regardless, the least amount of radiation burden for the patient should always be taken into account for each specific protocol.

Because of the higher radiation exposure to the patient from thallium-201, if other tracers are available they should be considered as alternatives, although the effective dose (mSv/MBq) has been revised and lowered by one-third from 2.1E−01 to 1.4E−01 25.

## Complementary techniques

Other noninvasive imaging techniques such as echocardiography, computed tomography, and MRI can provide useful information for both diagnostic and prognostic purposes in HF patients.

### Echocardiography

Transthoracic echocardiography remains the most performed myocardial imaging technique because of its widespread availability, low cost, and ability to provide information on valvular function, atrial size, right ventricular size, and contractility. Stress echocardiography, either with physical stress or with dobutamine, is extensively used to noninvasively detect the presence of CAD, the most common underlying etiology of DCM. In contrast, tissue Doppler imaging techniques 26,27 or, more recently, speckle-tracking echocardiography has been used to provide information on LV dyssynchrony before CRT, as well as to help in the prediction of response to this therapy 28,29. Echocardiography is the first imaging test recommended in the ACC/AHA guidelines 30,31 and in the ESC guidelines for the diagnosis of LV systolic dysfunction 32.

However, it is important to point out the need for acceptable echocardiographic windows to precisely determine these parameters, as well as to emphasize that the technique is less reproducible than nuclear cardiology techniques, requiring a considerable level of expertise of the echocardiographer.

### Coronary computed tomography

Coronary computed tomographic angiogram (CCTA) allows the noninvasive visualization of coronary anatomy and helps to define plaque morphology. It is currently considered a well-established tool for evaluating coronary disease 33,34. The main strength of CCTA seems to be the reliable exclusion of atherosclerotic disease because of its very high negative predictive value, which is particularly important in patients with intermediate-to-low CAD pretest probability. Thus, CCTA could be a useful tool in excluding CAD as the likely culprit for LV systolic dysfunction in this setting.

Coronary calcium evaluation is a useful way to noninvasively assess atherosclerotic burden and provides some guidance to the clinician for the differential diagnosis between a possible ischemic versus nonischemic etiology for LV systolic dysfunction 35. Nevertheless, further studies with larger series of patients are needed to establish its role in this group of patients.

It is important to point out that the information offered by CCTA is mainly anatomical and does not include data regarding the presence or absence of ischemia, which is of particular interest in case of patients with CAD who need a revascularization procedure. Nuclear techniques are very well standardized in this setting, offering valuable information on the presence and extension of ischemia, as well as on the status of the intraventricular synchronization, very useful in the case of patients evaluated for CRT.

### Cardiac magnetic resonance

Cardiac magnetic resonance (CMR) is a versatile tool used to assess etiology in congestive heart failure because of its capability for tissue characterization and simultaneous assessment of LV function and wall motion and the possibility of myocardial viability detection through the late enhancement gadolinium images, which accurately delineate a scar, a powerful marker of poor prognosis in DCM. CMR has the advantage of not using ionizing radiation. The American College of Cardiology and the American College of Radiology have recommended CMR as an appropriate tool for the evaluation of LV systolic dysfunction of unknown etiology 36. However, it is a very expensive technique that is not broadly available in cardiac services, contrary to the already established nuclear techniques.

## Key points

On the basis of the current available evidence, the following points represent our consensus position for guidance on the use of nuclear cardiology techniques for the assessment of HF and associated myocardial disease:

1. To assess ischemia, a 1- or 2-day rest–stress protocol probably with pharmacologic stress and nitrates for rest should be used. Assessment of viability is important and should include assessment of scar burden. Scar burden is defined by the total amount of scar in the LV expressed as a percentage of the total LV and is an independent prognostic factor as well as a variable for treatment choice. If ^18^F-FDG is available for viability assessment it should be used, but in most situations an adequate SPECT MPI can provide the necessary information.
2. In HF patients, gating to help measure function is very important, although these patients have more gating problems 37. Only a nongated MPI should be acquired in patients with atrial fibrillation if rejection of bad beats is not possible. QC of the R wave triggering is important. If possible use the R-R window to reject bad beats. Availability of backward gating will also improve the accuracy of assessment of the diastolic parameters.
3. Physicians should be aware of the differences in normal values for the functional parameters of the software being used. Specifically, the report should include assessment of LVEF, LV end-systolic volume, transient ischemic dilation, eccentricity, and LV dyssynchrony. In addition, in patients being assessed for CRT the report should also include the last viable segment to contract (Tables 1 and 2).
4. LV synchrony assessment is important, particularly for patients being considered for CRT therapy 13,14. LV synchrony may be assessed with either the rest or the stress study, preferably using the study with the most counts 27. Synchrony should be similar for the poststress and rest study if the stress acquisition is performed at least 1 h after stress 38. However, if image acquisition is performed closer to stress then differences in synchrony can be found in CAD patients 39,40. Gating should be done with either 8 or 16 frames per cardiac cycle. All acquisition parameters used should be the same as standard MPI acquisition 7. In processing, care must be taken to QC the selection of the base throughout the cardiac cycle as improper selection will lead to measurement errors. The processing software used should have been validated for the synchrony parameters used for evaluation. At this time the SD and histogram bandwidth of the LV phase histogram have been shown to be useful in determining dyssynchrony 13,14.
5. It has been shown that 71% of HF patients who fulfill the CRT criteria respond to the treatment if their bandwidth is greater than 135° and/or SD is greater than 43° 41. It has also been reported that just having this amount of dyssynchrony is not enough for a patient to have a positive response to CRT 17. The following is also important to assure a higher probability of success to therapy: (i) the patient should have less than 50% of scar burden 42, and (ii) the LV lead should be placed (if technically possible) in the last viable segment to contract.
6. Segmental time of onset of contraction may be evaluated either visually from dynamic phase displays or preferably through segmental phase histogram measurements.
7. Planar gated blood pool images have also been used for assessment of dyssynchrony 43–45. The advantage of this approach is that interventricular dyssynchrony can also be assessed, but the disadvantage is that it is a volumetric approach but not three dimensional. This limitation may be corrected with SPECT techniques of blood pool imaging, which are inherently three dimensional.
8. Echocardiography is another option for evaluating LV dyssynchrony, particularly using the appropriate software (three-dimensional speckle tracking) 29,46.
9. ^123^I-mIBG is commonly imaged in an anterior planar view initially (15–30 min after injection) and in delayed images 4 h later, and optionally with SPECT imaging 19. Global cardiac uptake is expressed as H/M ratio on the delayed images. With current imaging methods bad prognosis in HF patients is observed when H/M ratio is under 1.6 in delayed images; normal values are above this cutoff (Table 3). Tracer washout between initial and 4 h delayed images may also be measured, as well as the extent and severity of regional defect(s) on delayed tomographic imaging.

**Table 1:**
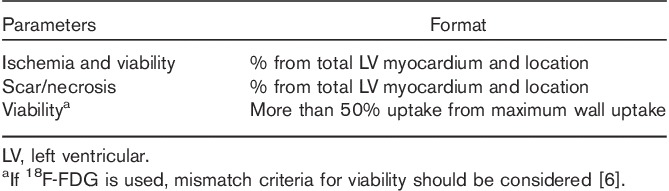
Perfusion parameters that should be reported in myocardial perfusion imaging studies of heart failure patients

**Table 2:**
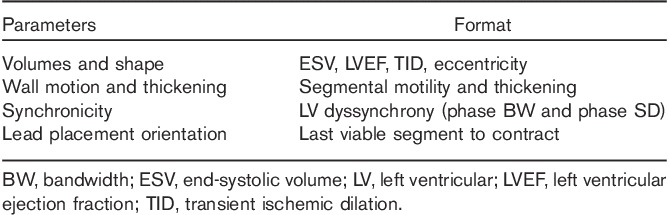
Functional parameters that should be reported in myocardial perfusion imaging studies of heart failure patients

**Table 3:**
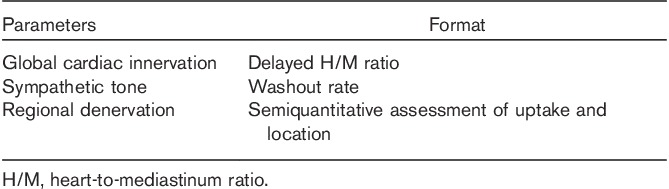
Parameters that should be reported in heart failure patients ^123^I-mIBG studies
